# Moral Hard‐Wiring and Moral Enhancement

**DOI:** 10.1111/bioe.12314

**Published:** 2017-03-16

**Authors:** Ingmar Persson, Julian Savulescu

**Keywords:** moral enhancement, moral bioenhancement

## Abstract

We have argued for an urgent need for moral bioenhancement; that human moral psychology is limited in its ability to address current existential threats due to the evolutionary function of morality to maximize cooperation in small groups.

We address here Powell and Buchanan's novel objection that there is an ‘inclusivist anomaly’: humans have the capacity to care beyond in‐groups. They propose that ‘exclusivist’ (group‐based) morality is sensitive to environmental cues that historically indicated out‐group threat. When this is not present, we are inclusivist. They conclude that moral bioenhancement is unnecessary or less effective than socio‐cultural interventions.

We argue that Powell and Buchanan underestimate the hard‐wiring features of moral psychology; their appeal to adaptively plastic, conditionally expressed responses accounts for only a fragment of our moral psychology. In addition to restrictions on our altruistic concern that their account addresses – such as racism and sexism – there are ones it is ill‐suited to address: that our concern is stronger for kin and friends and for concrete individuals rather than for statistical lives; also our bias towards the near future. Hard‐wired features of our moral psychology that are not clearly restrictions in altruistic concern also include reciprocity, tit‐for‐tat, and others. Biomedical means are not the only, and maybe not the most important, means of moral enhancement. Socio‐cultural means are of great importance and there are currently no biomedical interventions for many hard‐wired features. Nevertheless research is desirable because the influence of these features is greater than our critics think.

## Introduction

In a series of papers^1^ and a book^2^ we have argued that there is an urgent need to pursue research into the possibility of moral enhancement by biomedical means – e.g. by pharmaceuticals, non‐invasive brain stimulation, genetic modification or other means of directly modifying biology. The present time brings existential threats which human moral psychology, with its cognitive and moral limitations and biases, is unfit to address. Exponentially increasing, widely accessible technological advance and rapid globalization create threats of intentional misuse (e.g. biological or nuclear terrorism) and global collective action problems, such as the economic inequality between developed and developing countries and anthropogenic climate change, which human psychology is not set up to address. We have hypothesized that these limitations are the result of the evolutionary function of morality being to maximize the fitness of small cooperative groups competing for resources. Because these limitations of human moral psychology pose significant obstacles to coping with the current moral mega‐problems, we argued that biomedical modification of human moral psychology may be necessary. We have not argued that biomedical moral enhancement would be a single ‘magic bullet’ but rather that it could play a role in a comprehensive approach which also features cultural and social measures.

This proposal has met with widespread opposition,^3^ even from those who enthusiastically embrace biomedical enhancement of other human capacities than moral ones.^4^ We have responded to many of these criticisms.^5^ In a recent provocative and erudite argument, Russell Powell and Allen Buchanan raise a new kind of challenge.^6^ They correctly point out that we believe that ‘cultural forms of moral enhancement (e.g. moral education) have been only moderately effective and are simply not up to the task of mitigating major anthropogenic harms and existential risks.’^7^ But, they argue, we have seriously underestimated the power of such means of moral enhancement, and the amount of moral progress that the human species has achieved by their means. They hypothesize that it is socio‐cultural, not biomedical, means of moral enhancement that offer the best prospect for addressing the problems humans currently face.

They accuse us of having exaggerated the constraints that evolution has placed on moral progress. They think that in line with prevailing evolutionary accounts of morality, we have failed to take account of an ‘inclusivist anomaly,’ that is, the capacity of humans to extend their pattern of care and concern beyond in‐groups, which Peter Singer called the ‘expanding circle’.^8^ They write: ‘In particular, we propose that ‘exclusivist’ (parochial, group‐based) morality is the result of an adaptively plastic (conditionally expressed) moral response that is sensitive to environmental cues that were historically indicative of out‐group threat’.^9^ In summary, when there is an out‐group threat, we become exclusivist. When there is no perceived out‐group threat, we are inclusivist. Their conclusion is that when this inclusivist anomaly is appreciated, ‘it becomes clear that BME [biomedical moral enhancement] technology, as narrowly conceived by BME proponents, is unlikely to be necessary or effective.’^10^ Socio‐cultural innovations that alleviate ‘infectious disease, resource scarcity, physical insecurity, interethnic conflict and low rates of productivity’ suffice to foster inclusive morality.^11^

## The General Outline of the Dispute

Although we are in agreement with much of Powell and Buchanan's argument, it is necessary to respond to it because there are some crucial respects in which we believe them to be mistaken both about the position we adopt and about the extent of ‘hard‐wired’ moral traits – i.e. genetically based traits that manifest themselves in normal social conditions – in human nature and, consequently, of the role biomedical moral enhancement, BME, could play in the future of humanity. A common point of departure between us is that human morality and moral psychology have been shaped by evolutionary factors. The gist of this account is nicely provided by Powell and Buchanan:^12^

The prevailing evolutionary explanation of morality holds, in brief, that morality evolved via natural selection in small to moderate‐sized hunter‐gatherer groups because it promoted higher levels of within‐group cooperation. It did this by reducing free‐riding and mitigating selfish tendencies… This increase in cooperation gave groups with effective moralities several fitness advantages over non‐moral and less moral ones.

The question is to what extent this origin makes the moral mind of human beings ill‐suited to cope with the moral mega‐problems that face them today, problems of increasing availability of weapons of mass destruction, anthropogenic climate change and environmental degradation, and a huge global inequality of welfare. Powell and Buchanan's conclusion is that ‘cultural innovations that make use of our best understanding of the evolutionary development of human morality stand the best chance of meeting … [our] ethical goals’.^13^ They relegate BME to a much more modest place than they hold us to assign to it.

Like their designated adversaries –‘evoconservatives’ and ‘evoliberals’ – they believe that, originally, human morality was quite ‘exclusivist’ because it evolved to facilitate in‐group cooperation in order to make these groups more effective in the competition with other groups over scarce resources in a hostile environment. As already indicated, they propose however that this exclusivist or ‘(parochial, group‐based) morality is the result of an adaptively plastic (conditionally expressed) moral response that is sensitive to environmental cues that were historically indicative of out‐group threat’.^14^ They take this to mean that if the threat from out‐groups disappears, morality will tend to develop in an inclusivist direction, by rejecting:

group‐based restrictions on membership in the moral community, such as those based on race, ethnicity, gender, species, or the self‐serving cooperative relationships between groups, and that avoid arbitrary discounting of the interests of the members of out‐groups.^15^

By contrast, both evoconservatives and evoliberals are portrayed as taking evolution to place fixed constraints on morality that are incompatible or in tension with such an inclusivist ‘shift’ or ‘anomaly’.^16^ The defining difference between the evoconservative and the evoliberal camps is that evoconservatives regard evolutionary constraints as insurmountable or normatively guiding, whereas evoliberals regard them as so hard to overcome that ‘the most effective means of transcending our inability to extend moral concern beyond the group, including to individuals of future generations, is not sociocultural but biological, in the form of BME’.^17^

But Powell and Buchanan also mention a ‘weaker evoliberal claim, which holds simply that our moral problems are so momentous that any possible means for reducing our parochial tendencies should be on the table, BME included’. They go on to say that they

are happy to embrace this weaker evoliberal claim, but note that it is not a very interesting argument since it does not make any substantive assertions about how important BME is likely to be given our evolutionary history, and thus it does not say anything about how BME should be prioritized relative to ‘traditional’ modes of moral enhancement.^18^

Now, it is in fact this weaker evoliberalism that corresponds most closely to our own position. In reply to other critics,^19^ we have emphasized that our BME proposal is ‘cautious’ rather than ‘confident’. Whereas a confident proposal would claim precisely that ‘the most effective means’ of moral enhancement are biomedical, a cautious proposal claims only that it is likely to be one indispensable means among others. Thus, we are in agreement with Powell and Buchanan when they write:

we think it is reasonable to view biomedical intervention as one potential instrument in our diverse moral enhancement toolkit. Like many ethicists writing on this topic, we see no in‐principle objection to using biomedical technologies in conjunction with cultural modes of moral enhancement to bring moral motivations and behaviors in line with the norms we have come to endorse.^20^

As we shall see, however, the fact that we acknowledge other means of moral enhancement alongside BME undercuts many of their qualms about BME. Since BME research is in its infancy, we regard it as too early to judge how effective a means of moral enhancement it could be relative to other means. We believe it is premature to assert, as they do, that ‘cultural innovations stand a far better chance’ of ‘reducing the incidence and intensity of intergroup conflicts’.^21^

We do not agree, however, that this refusal to rank BME relative to other means of moral enhancement reduces a weaker evoliberalism to a position that is not ‘very interesting’. First, as not least the reception of our work testifies, there are a number of writers who deny that BME could or should play *any* role in the moral enhancement process – even writers, like John Harris,^22^ who eagerly endorse biomedical methods in the case of cognitive enhancement. Secondly, we wonder whether Powell and Buchanan are consistent when they write that they are ‘happy to embrace’ weaker evoliberalism, for they write in the context of a quotation from our work that evoliberalism affirms ‘a degree of ‘moral hardwiring’ that is significantly determinative of the shape of human morality, robust against changes in the moral developmental environment, largely insensitive to rational persuasion’.^23^ In what follows we will contend that they are unclear or ambiguous about the degree to which such hard‐wiring is determinative of our morality or moral psychology, but that in any case they underestimate it.

## What is the Purport of Powell and Buchanan's ‘Moral Inclusivism’?

To illustrate their idea that ‘exclusivist moral psychology is an adaptively plastic trait’, they compare it to the elaborate armour some species of water fleas develop ‘only if they detect the chemical correlates of a predator in the water in which they grow’.^24^ In the case of exclusivist moral psychology ‘the relevant threat relates to out‐group predation and competition’.^25^ Consequently, ‘exclusivist tendencies will … be tempered in environments in which out‐group threats are not detected during development’.^26^ These ‘environments that are favourable to inclusivist morality are rare, having emerged only recently in human history and remaining largely confined to well‐resourced populations’,^27^ but they are present in the affluent societies of the West. Thus, ‘inclusivist morality is a luxury good’.^28^ It is a good because, like flea armour, exclusivist morality has costs, since ‘out‐group aggression, antipathy, and distrust reduce the chances of mutually beneficial interactions with neighbouring groups, such as trade and alliances.’^29^

Powell and Buchanan maintain that the morality of Western societies is inclusivist to the point of including even nonhuman animals: ‘nonhuman animals are now widely regarded as proper subjects of moral worth’. They refer to ‘domestic and supranational legal regimes governing animal cruelty’^30^, but such legal regimes are lacking in many countries, and in some countries in which they do exist, they leave much to be desired. For instance, domestic US legislation redefines “animal” to exclude birds, rats and mice bred for research. The strongest support of their claim is probably the EU supranational legislation, which declares that ‘since animals are sentient beings [Member States] shall pay full regard to the welfare requirements of animals’ (Treaty on the Functioning of the European Union, 2009, art. 13).

However, rather than being a counter‐instance to our view that inclusivist or egalitarian ideologies are often a ‘façade’ covering underlying exclusivist or xenophobic motivation, this is a perfect illustration of the kind of rift between official moral declarations and actual practice that we have in mind.^31^ Nobody could seriously claim, for instance, that when billions of nonhuman animals are bred and killed for food in the EU, this practice is in accord with its lofty legal principle about ‘full regard to the welfare requirements of animals’.

Apparently aware of this sort of objection, Powell and Buchanan concede that ‘nonhuman animals are still treated as mere units of production’. Their reply is that ‘these facts do not belie this important inclusivist trend any more than the fact that many countries still deny basic civil and political rights undercuts the global trend in the expansion of basic liberties’.^32^ But this reply conflates to two distinct issues: how widespread a normative trend is and the extent to which it is implemented in places to which it has spread. Norms concerning animal welfare are neither globally widespread, nor effectively implemented.

This distinction is also relevant to another of their examples of the current inclusivist morality: a ‘culture of human rights’ to the effect that ‘all human beings are entitled to be treated in ways that respect their basic interests regardless of which ethnic, racial, or religious groups they happen to belong to, and regardless of their age or able‐bodiedness’.^33^ As Powell and Buchanan admit, this doctrine of equal respect is far from having achieved worldwide coverage, but even among the affluent liberal democracies in which it is officially cherished, only a few have reached the modest goal set by the United Nations decades ago of giving 0.7% of the GDP to alleviate the present enormous global inequality in respect of welfare. In fact, the gap between the richest and the poorest communities is constantly growing: the poorest 80% of the Earth's population now account for less than 10% of the global wealth, and the 80 richest *individuals* own as much the poorest 50%. Again, there is a glaring discrepancy between saying and doing.

The concern for animal welfare and the ‘culture of human rights’ that Powell and Buchanan refer to as examples of ‘sweeping, progressive moral change’^34^ are part of something that could be called the ‘official’ or ‘public’ morality of some liberal democracies. This is the morality that comes to expression in their laws, in declarations of their leading politicians and the media. The official morality of Western democracies is broadly egalitarian: it ascribes equal worth or value in some sense to all human beings. It also endows non‐human animals with some moral status, though not as high as that of humans. The official morality should be distinguished from a more intellectually reflective or philosophical morality. The latter is not anything unified; it rather comprises important controversies, but what is particularly noteworthy for present purposes is that it is in considerable tension with the official morality with respect to human equality.

Although Powell and Buchanan sometimes speak as though inclusivist morality implied that all humans have equal moral worth^35^ it should be noticed that from the rejection of the moral relevance of *some* differences between people, like race, ethnicity or gender, it doesn't follow that *no* differences can be the ground for moral differentiation. They hold that inclusivist shifts ‘are premised on a ‘subject‐centred’ or non‐strategic conception of basic moral status’.^36^ But such a conception fits well with taking differences in intelligence, rationality or morality to justify differences in moral worth, since they are characteristics of subjects (of consciousness). These characteristics are frequently adduced as grounds for elevating humans above non‐human animals, but humans also differ as regards them, so they are unpromising as foundations for equal human worth or moral status. In fact, to justify the idea of an equal worth or moral status of *all* humans above that of *all* non‐human animals is philosophically very problematic. This means that the jury is still out on the issue of whether this article of the official morality can count as moral *progress*. By contrast, it is much more certain that grounds such as race and gender are indefensible.

## Moral Hard‐Wiring Presupposed by the Powell‐Buchanan Model

Let us, then, leave unsettled what is the precise import of their inclusivist shift/anomaly. The point we would like to make is that we do not propose that there are any significant evolutionary constraints on where this shift/anomaly can take us in so far as this refers to official or intellectually reflected morality. It follows that evolutionary explanations cannot completely account for human morality; it is also formed by socio‐cultural factors and reasoning, or ‘open‐ended normativity’ in Powell and Buchanan's vocabulary.^37^ But while evolutionary considerations cannot explain these cognitive or doxastic elements of morality, we do believe that they can explain a number of *motivational or non‐cognitive* elements of our moral psychology, and why they can have a hard time catching up with the former elements. Powell and Buchanan grossly underestimate the number of these motivational or non‐cognitive elements, as we will now argue.

Remember, their idea is that ‘exclusivist moral psychology is an adaptively plastic trait’ like the elaborate armour some species of water fleas develop ‘only if they detect the chemical correlates of a predator in the water in which they grow’.^38^ A first shortcoming of this hypothesis is that it doesn't explain all forms of moral exclusivism or partiality. Consider the so‐called *agent‐relative prerogatives or permissions* proposed e.g. by Samuel Scheffler to the effect that we are allowed to favour ourselves and those near and dear at the expense of others.^39^ The flea armour analogy does not account for such permissions because their source need not lie primarily in the threat from some out‐group. Rather, it lies in our altruistic concern, benevolence, or sympathy being ‘stratified’, having several layers or grades: we tend to be concerned about people in proportion to how much we are exposed to and cooperate with them in mutually beneficial ways. The people we are most exposed to in these ways include close kin, like children and siblings; thus, they belong to those for whom we care most. We are virtually indifferent towards strangers, even if co‐operation with them occurs at some international level, so we would do very little to help them out. Between these extremes, there are various groups of individuals with whom we associate in some circumstances and for whose welfare we have a smaller degree of concern. This is what we meant when we have spoken of our concern or altruism as limited and parochial.

Now we find it plausible to regard this is pattern of concern as a piece of ‘moral hardwiring’ that is significantly determinative of the shape of human morality, robust against changes in the moral developmental environment, largely insensitive to rational persuasion’, in the words of Powell and Buchanan. Rational persuasion or cultural pressures are unlikely to make parents impartial between their own children and other children – to the chagrin of some social utopists from Plato to present‐day communists. Thus, their hypothesis that ‘exclusivist moral psychology is an adaptively plastic trait’ sits ill with this stratification of our concern or altruism. This oversight is remarkable, since they talk about parochial altruism that is part of our evolutionary legacy.^40^ Surely, they can't deny that it is a piece of biological or evolutionary hard‐wiring that we are disposed to be most concerned about close kin.

It might be objected that agent‐relative permissions are not ‘exclusivist’ strictly speaking, since they don't altogether *exclude* anyone from moral concern; they merely allow a reduction of concern for some to the benefit of others. This is a reflection of the pattern stratified or parochial concern that underlies them. But despite the term, Powell and Buchanan occasionally take exclusivism to amount to ‘discounting’.^41^ And, surely, features like race or gender may not altogether exclude concern, either, but only reduce it. Such a reduction of concern is sufficient to explain why, when certain resources become scarce, some individuals are excluded from a share of them. In this way, the pattern of stratified concern is indispensable in order to understand the operation of the exclusivist mechanisms they emphasize.

So the picture that is emerging is this. Individuals within the field of stratified concern can be grouped together in various ways, on the basis of conspicuous features, like colour of skin, the way they dress, the language they speak, religious affiliation, and so on. Humans are, as Powell and Buchanan remark, wired up to categorize individuals on the basis on various easily observable features. These features are ‘translucent’, that is, they tend to make it harder for us to sympathize and identify with the beings who ostensibly belong to a different group than with people more like us – harder, but not impossible. If we regularly have dealings with these ‘outliers’ to mutual benefit, this resistance can be overcome. On the other hand, if resources grow more scarce, or if there turns out be a risk that they carry diseases and, thus, start posing threat to us and those who are near and dear to us, we readily develop hostility towards them. This hostility tends to strengthen the concern we have for those near and dear whom we are likely to need to cooperate with to fend off the ‘intruders’. In other words, exclusivist mechanisms of the kind put forward by Powell and Buchanan, which have been inoperative in more favourable conditions, come into operation.

However, what they overlook is that even in more favourable conditions when a community prospers and is under no external threat, exclusivism can and does kick in. As remarked, humans are inclined to be especially partial to the small group of close kin and dear friends. This is what is called *nepotism and cronyism* when it is excessive in some fashion. It thrives even under conditions of luxury and excess, such as those of courts and palaces, as well as the most affluent societies. It exists because the *best* things in life are by necessity scarce: the greatest power, the highest status, and the most attractive mates. It follows that if a community is not threatened from *without*, by external adversaries, competition *within* the community between different clans or families for these precious things will come to the forefront.

Notice how dissimilar nepotism and cronyism are to racism, sexism and speciesism: whereas the latter forms of discrimination turn on differentiation grounds that the inclusivist shift discards, the same cannot be true for the rejection of nepotism if there are agent‐relative permissions to treat friends and family in privileged ways, as some moral philosophers affirm, since their differentiation grounds – kinship and friendship – would then be morally relevant. Rather nepotism and cronyism consist in some sort of ‘overworking’ of these grounds. The cause of such overworking is hardly always ‘harsh conditions’^42^ that are removable by social reform, as Powell and Buchanan contend; it is sometimes an immoderate degree of concern.

There are then two connected reasons, of importance in the present context, why their flea armour analogy is misguided. What elicits exclusivism is not a single, fixed stimulus as the chemicals released by predators that is present in some, but not all, circumstances; it is such a large number of factors that one or another of them is bound to be in play at any time. While some of them come into play only in the ‘harsh conditions’ of famines and epidemics and so on, others are at work even under conditions of prosperity. Then it becomes apparent that another cause of exclusivism is *the stratification of human concern*. It is important to highlight this factor for present purposes, since it is surely a product of evolution. Its upshot, equally important to highlight is, contrary to what Powell and Buchanan assert, *the ubiquity of competition between groups for some kind of resource or other, and the ubiquitous presence of some exclusivist triggers*. This concords with their remark that, according to ‘the prevailing evolutionary explanation of morality’, the ubiquity of group competition and ‘parochialism’ are necessary for ‘the evolvability of human altruism’:^43^ the trait of altruism will only be selected for if it increases the reproductive fitness of some in‐group in its struggle against other groups. This should dampen their optimism about the possibility of rooting out all exclusivism by social and institutional means. Certainly, we should do what we can to combat exclusivism by these means, but a society without nepotism, say, seems utopic, at least as long as we lack biomedical methods to assist us in tackling it.

We see, then, the underlying stratified concern as one target of bioenhancement, but it is misleading to present us, as Powell and Buchanan do, as believing that ‘we are likely to discover biomedical interventions that strengthen prosocial attitudes and behaviours toward strangers and out‐groups’.^44^ We do not believe that currently available ways of modifying pro‐social concern, such as drugs like oxytocin, would lead to a bioenhancement of altruistic concern that would diminish its stratified character and make our concern for those near and distant to us more equal. Perhaps reduction in the statification of altruistic concern will not happen unless we find ourselves in socio‐economic conditions of the sort our critics stress, and become intellectually convinced by moral reasoning that certain conspicuous group divisions lack moral significance. However, if the altruistic concern of many of us is not intensified, we do not believe that it will reach outliers, even if the barrier between them and us is demolished or becomes less ‘opaque’. If the strength of concern did not matter, even psychopaths could be made to care about others by the sociocultural means favoured by Powell and Buchanan. Therefore, we think it is important to increase the capacity for altruistic concern of most of us, so that it gets closer to the concern of those of us who are most concerned, and we have argued that this may be most effectively done by future biomedical means which seem within reach.

We are then well aware that our altruistic concern ‘is tightly bound to partiality’ and that, therefore, merely boosting the capacity for it ‘can lead to a range of poor moral decision‐making’.^45^ We think that this has to be kept in check by moral education of a traditional sort, as well as the socio‐cultural and institutional means that Powell and Buchanan advocate. We may be in need of biomedical means to be completely successful in this endeavour, since the partiality or stratification of our concern is surely hard‐wired, but no such means to fight its stratification, as opposed to its strength, are on the horizon as yet. They write: ‘the problem boils down not to a general deficit of concern but rather to the fact that concern is not directed in the right ways (e.g. felt in response to the disregard of the interests of out‐group members)’.^46^ We believe, on the contrary, that there is *both* a general deficit of concern in the case of many of us, and misdirections of the available quantum of concern to contend with. It is highly unlikely that the level of altruism or empathy that evolution selects is optimal for the goals we have now set ourselves.

## Moral Hard‐Wiring Beyond the Reach of the Powell‐Buchanan's Model

However, we are not yet at the end of the list of hard‐wired features of our moral psychology that Powell and Buchanan overlook. When exemplifying the ‘group‐based restrictions on membership in the moral community’, they mention ‘self‐serving cooperative relationships between groups’.^47^ This should include not merely groups that we refuse to cooperate from the start, but also groups and individuals with whom we discontinue cooperation because they have not reciprocated properly, that is, free‐riders and defectors. As we point out,^48^ *the tit‐for‐tat strategy* is built into us, i.e. we are prone to react with gratitude to those who return our favours, a feeling that they deserve to be benefited in turn, and with anger or a feeling that they deserve to be punished to those who do not. Since the latter reaction incorporates excluding individuals from further cooperation, it seems to qualify as exclusivist.

We do not see, however, how the exclusion of defectors and free riders fits into Powell and Buchanan's scheme, since they describe the items on their exclusivist list – items like race and gender – as involving ‘arbitrary discounting’^49^ of interests, but the desire to punish free‐riders and defectors does not appear to be ‘arbitrary’. Nor does punishment of wrongdoers.

Let's continue down the list of candidates for the status of being hard‐wired. We have argued that Powell and Buchanan's flea armour model cannot account for such forms of exclusivism as nepotism or cronyism, or tit‐for‐tat. But nor can it account for the fact that ‘we tend to discount the interests of future generations in deciding how we will interact with the environment’.^50^ This discounting is obviously important for the problem of mitigating anthropogenic climate change which, as they rightly point out ‘also has inclusivist moral dimensions’.^51^ We have hypothesized that this discount is partly due to what we called *the bias towards the near future*:^52^ we are spontaneously more concerned about positive and negative events in the nearer than in the more remote future. This bias is not strictly a moral bias because it is in operation in the domain of self‐interest or prudence as well, that is, when we concerned with the impact of events solely on our own interests, but it is also morally relevant, i.e. relevant to the treatment of others.

Now notice that this bias does not fit Powell and Buchanan's characterization of an adaptively plastic response which is sensitive to environmental conditions like out‐group threat: obviously, we are not less concerned about our future selves or future generations because they pose threats to us. More likely, the bias towards the near is a biologically hard‐wired reaction which is ‘robust against changes in the… developmental environment,  [and]  largely insensitive to rational persuasion’. Even if we are intellectually persuaded that the fact that, say, something is postponed until tomorrow by itself is not anything that makes it matter less, we cannot easily shake off a feeling to the contrary. Plausibly, we possess this temporal bias because it has served our reproductive fitness: the threats most urgent to deal with are generally those in the imminent future.

In so far as the fact we are dragging our heels in coping with anthropogenic climate change is put down to the bias towards the near, then, it is dependent on a hard‐wired feature. But another factor underlying this procrastination is also plausibly hard‐wired. Powell and Buchanan observe that we are prone to ‘favouring the lives of concrete individuals over “statistical” lives’.^53^ While many of us are quite capable of sympathizing with individuals suffering before our very eyes, we respond rather indifferently to verbal reports of the suffering of unknown individuals. Moreover, as we have noted^54^ our capacity to sympathize with sufferers is not proportional to their number: it does not grow as the number of sufferers does; in fact, the suffering of a single concrete individual can arouse as much sympathy as masses of sufferers before our eyes, let alone ones that are merely known by description. Therefore, although millions may suffer because of climate changes, this leaves us largely unmoved for the reason that they are statistical and in the remote future.

The hard‐wired limitations of our sympathy or altruism as regards number and concreteness are also pertinent to our handling of another moral mega‐problem: global economic inequality. But here another form of exclusivism kicks in as well. Powell and Buchanan talk as though moral enhancement was merely a matter of a transition from exclusivism to inclusivism by breaking down barriers like racism, sexism and speciesism. But, as we point out,^55^ alongside these limitations of our altruism, *the act‐omission doctrine* – i.e. the doctrine that harming is harder to justify morally than omissions to benefit – is surely part of the explanation of why affluent nations have done so little to reduce the poverty of developing countries, although the issue has been on the UN agenda for about half a century. This doctrine also represents moral exclusivism of a sort: not by excluding any type of individuals from the ambit of morality, but a kind of behaviour: omissions. The adoption of an inclusivist morality does not make much progress towards solving the moral mega‐problem of the global welfare inequality if it is coupled with the view that what this morality requires is chiefly a matter of refraining from causing harm, and recommends little in the way of positive benefiting.

Now it is admittedly philosophically controversial whether the act‐omission doctrine is untenable, but those who are convinced that it is – including the present authors – have experienced that it is not easy to shake off the feeling that we are less responsible for what we let happen than for what we actively cause. We have speculated that this is due to the fact that our primordial sense of responsibility is firmly based on causality.^56^ But irrespective of whether this is on the right track, it seems hard to deny that the attitude which manifests itself in the act‐omission doctrine is partly hardwired and quite resistant to rational argument.

Linked to the act‐omission doctrine is another attitude that we have proposed is hard‐wired: that there are *property rights* to the effect that we have a right to what we are the first to occupy or appropriate of unowned natural resources, and what we manufacture out of these resources by our own labour.^57^ Property rights help to underpin the idea that we are permitted to omit helping others by giving them things we own, though they need them much more than we do. They help explain how we can accept a world in which, for instance, the 80 richest *individuals* own as much the poorest 50%.

We do seem to carry around a large repertoire of evolutionary hardwired attitudes which are quite insensitive both to changes in the social environment and to rational argument. Some of them, on which we have focused so far, are moral, or morally relevant, but others are non‐moral. Non‐moral examples would be various phobias, like arachnophobia, which still afflicts people, though their ancestors have lived for hundreds of generations in territories in which spiders are not dangerous, and they are well aware of this fact, or the craving for sugary and fatty food which is causing increasing obesity in industrial parts of the world. We believe that just as biomedical treatment is liable to be an effective intervention in these cases, it may provide effective help in overcoming moral shortcomings due to the factors reviewed above.

## Concluding Remarks

Powell and Buchanan claim that ‘there are effective institutional solutions to the problems of intergroup conflict that drive many of the moral catastrophes that rightfully worry evoliberals’^58^ and that ‘none of these cultural innovations require intervening at the level of individual moral capacities’.^59^ There are doubtless *conceivable* international agreements that would solve the problems of anthropogenic climate change and global poverty. Some of them have been on the table of UN led negotiations for decades, but all the same, they have not won wider acceptance and been implemented. Why is that? The short answer would seem to be that the current generations of people in affluent countries and in countries on the way to affluence, like China, are not sufficiently morally motivated to undertake sacrifices of welfare for the sake of others. Above we have listed several hard‐wired features that contribute to making this fact comprehensible.

They also remark that ‘the threat of punishment exerts a much stronger influence on prosocial behaviour than oxytocin or other BME variables’.^60^ This is not anything we have to deny. But if they find that the role we assign to BME as one means of moral enhancement among others not ‘very interesting’, we are obliged to retort that it is not ‘very interesting’ to highlight punishment which has been used effectively for millenia. Furthermore, with respect to the moral mega‐problems of primary concern to us we believe that, without a moral change of their attitude, a majority of voters in affluent democracies are unlikely to support governments which impose on them laws that on pain of punishment force them to make sacrifices for the benefit of the globally poor and future generations. So to their claim: ‘Institutions can encourage people to act as if, for example, foreigners or domestic minorities are of equal moral worth, even if they are incapable of loving or empathizing with them’,^61^ we reply: yes, but in democracies the problem is to get voters to elect politicians who propose such institutions.

Powell and Buchanan also maintain that ‘most moral catastrophes are rooted in intergroup conflict’.^62^ But this does not seem an apt characterization of the mega‐problems of climate change and global inequality. With respect to them, we want to remove the word ‘not’ in their claim that ‘efforts to achieve major moral progress and avert serious moral catastrophes do not go against the human evolutionary grain *tout court*’.^63^

This is not to deny their claim that there has been an ‘expansion of inclusivist morality over the last couple of centuries’^64^, especially after World War II. But it should not be assumed that this expansion cannot be reversed if climate change and population growth lead to a shortage of vital resources like water and arable land, and bring hordes of climate refugees to the doorstep of the affluent nations – that is, undermine those ‘rare’ ‘environments that are favorable to inclusivist morality’. Recent lapses into brutality in, for instance, Nazi‐Germany and ex‐Yugoslavia, warn us against rashly concluding that human nature has changed to the core.^65^ Indeed, perhaps the growing nationalism in Europe and Donald Trump's slogan ‘America first’ are signs of the beginning of a major reversal.

Consequently, we think Powell and Buchanan are more optimistic than their arguments warrant. They might counter that we are overly optimistic in thinking that BME ‘interventions could be implemented on a sufficiently massive scale … and … with sufficient rapidity to address imminent catastrophic threats’.^66^ But this isn't anything that we are at all confident about; indeed, we are not even confident that safe and sufficiently effective means of biomedical moral enhancement will be found in the foreseeable future even if this research is adequately funded – which we are definitely not confident about. Our main point is simply that the human predicament is now so ominous that we should not spurn developing any means that contemporary science could produce that could significantly improve it. The fact that a world‐wide application of a form of biomedical treatment appears utopic is usually not seen as a decisive reason not to try to develop it. Indeed, the same can be said of their institutional reforms – these are hardly likely to touch all seven billion people alive and enable them to cooperate to solve the moral mega‐problems of our time.

The possibility of development of effective BME is something that our critics concede when they write, for instance, ‘we think it is reasonable to view biomedical intervention as one potential instrument in our diverse moral enhancement toolkit’.^67^ On the other hand, we endorse their belief in the efficacy of institutions’ and ‘cultural innovations’, along with the importance of moral enhancement by the traditional means of reasoning and example. All this is part of what we call a cautious BME proposal.^68^ In conclusion, there are two chief points we have tried to make against Powell and Buchanan:

1. They grossly underestimate the number of hard‐wiring features of moral psychology; once this is recognized, it is seen that their appeal to adaptively plastic and conditionally expressed response accounts for only a fragment of our moral psychology, and that it presupposes the operation of some hard‐wired features.
  In addition to the restrictions on our altruistic concern that their account is designed to deal with – such as racism, sexism and speciesism – there are ones it is ill‐suited to deal with, namely that this concern is stronger for kin and friends, for concrete individuals than statistical, is insensitive to numbers, and biased towards the near future. Then there are apparently hard‐wired features of our moral psychology that are not such restrictions: reactions involved in reciprocity and tit‐for‐tat, the stronger sense of responsibility for what we cause than what omit to prevent, or let happen, and the reactions associated with property rights.
2. They mistakenly take our defence of BME to be rather ‘exclusivist’, whereas it is in fact quite ‘ecumenical’: we do not claim that biomedical means are the most important means of moral enhancement, let alone the sole means. We acknowledge the importance of the socio‐cultural and institutional means they emphasize, along with input from our rationality. Additionally, we concede that there are as yet no available biomedical means to tackle many of the listed hard‐wired features, and there may never be, while insisting that it is desirable that there be such means because the influence of hard‐wired features is greater than our present critics think.

A further issue is which of the hard‐wired features reviewed are *correct or justifiable*. It is especially controversial whether those features which underpin agent‐relative permissions, the act‐omission doctrine, property rights and desert are justifiable. This problem also confronts the institutional type of moral enhancement Powell and Buchanan advance because it consists in ‘interventions external to the agent that generate behavior that is in accordance with correct moral principles, irrespective of its motivations’.^69^ What are these ‘correct’ moral principles? What is the purport of their moral inclusivism more precisely, does it for instance comprise the doctrine of the equal moral worth of humans earlier discussed? They must provide an answer to this question since their stated aim is to contribute to an ‘evolutionary developmental model of moral *progress*’.^70^ To some extent, we sidestep this problem by taking moral enhancement to consist in enhancement of moral *motivation*, e.g. motivation to do what you *think* is just and beneficial. We believe there is a considerable amount of consensus about what this is, which has constituted the basis of functioning human societies through history. But there are also significant disagreements that will have to be resolved by rational reflection if we are to be as fit as possible to cope with the moral mega‐problems of our time.

## Funding

Julian Savulescu's work on this paper was supported by the Wellcome Trust: WT104848/Z/14/Z

## Footnotes

1
I. Persson & J. Savulescu. The Perils of Cognitive Enhancement and the Urgent Imperative to Enhance the Moral Character of Humanity. *J Appl Philos,* 2008; 25(3): 162–177; I. Persson & J. Savulescu. Moral Transhumanism. *J Med Philos* 2010; 35(6): 656–669; I. Persson & J. Savulescu. 2011a. Unfit for the Future? Human Nature, Scientific Progress, and the Need for Moral Enhancement. In *Enhancing Human Capacities* J. Savulescu, R. Ter Meulen & G. Kahane, eds. Oxford: Wiley‐Blackwell: 486‐500; J. Savulescu & I. Persson. Moral Enhancement, Freedom and the God Machine. *The Monist* 2012; 95(3): 399‐421; I. Persson & J. Savulescu. Getting Moral Enhancement Right: The Desirability of Moral Bioenhancement. *Bioethics* 2013; 27: 124–131; I. Persson & J. Savulescu. Against Fetishism about Egalitarianism and In Defence of Cautious Moral Bioenhancement’, *Am J Bioeth* 2014a: 14:39‐42.

2
I. Persson & J. Savulescu, 2012. *Unfit for the Future*, Oxford: Oxford University Press.

3
N. Agar. Why is it possible to enhance moral status and why doing so is wrong? *J Med Ethics, *2013; 39: 67–74; E. Fenton. The perils of failing to enhance: a response to Persson and Savulescu. *J Med Ethics,* 2010; 36: 148–151; M. Hauskeller. 2013. *Better Humans?* Durham, NC: Acumen; R. Sparrow. Better living through chemistry? A reply to Savulescu and Persson on ‘moral enhancement'. *J Appl Philos* 2014; 31: 23–32.

4
J. Harris. Moral Enhancement and Freedom. *Bioethics*, 2011; 21: 102‐11. 10.1111/j.1467-8519.2010.01854.xPMC366078321133978

5
I. Persson & J. Savulescu. The Turn for Ultimate Harm: A Reply Fenton. *J Med Ethics* 2011b; 37(7): 441– 444; I. Persson & J. Savulescu. Should Moral Bioenhancement Be Compulsory? Reply to Vojin Rakic. *Journal of Medical Ethics,* 2014b; 40: 251–252. I. Persson & J. Savulescu. Unfit for the Future: Reply to Commentators. *J Med Ethics* 2015a; 41: 348–352; I. Persson & J. Savulescu. The Art of Misunderstanding Moral Bioenhancement, *Cam Q Healthc Ethics*, 2015b; 24: 48–57; J. Savulescu, T. Douglas, & I. Persson. ‘Response to Commentators'. in *Future of Bioethics: International Dialogues*. A. Akabyashi (ed.) Oxford: Oxford University Press: 131–138.

6
R. Powell & A. Buchanan, A. 2016. The Evolution of Moral Enhancement. In *The Ethics of Human Enhancement: Understanding the Debate*. S. Clarke, J. Savulescu (eds.) Oxford: Oxford University Press: 239–260.

7
Ibid, p. 239.

8
P. Singer. 2011. *The Expanding Circle: Ethics, Evolution, and Moral Progress*. Princeton: Princeton University Press.

9
Powell & Buchanan, *op. cit*. note 6, p. 240.

10
Ibid, p. 240.

11
Ibid, p. 247.

12
Ibid, pp. 240–1.

13
Ibid, p. 240.

14
Ibid, p. 240.

15
Ibid, p. 242.

16
Ibid, p. 240.

17
Ibid, p. 243.

18
Ibid, p. 243.

19
Persson & Savulescu, 2014a, *op. cit* note 1 & 2015b, *op. cit*. note 5.

20
Powell & Buchanan, *op. cit*. note 6, p. 258.

21
Ibid, p. 250.

22
Harris. *op. cit*. note 4; 21: 102–11.

23
Powell & Buchanan, *op. cit*. note 6, p. 243.

24
Ibid, p. 246.

25
Ibid, p. 246.

26
Ibid, p. 246.

27
Ibid, p. 247.

28
Ibid, p. 247.

29
Ibid, p. 246.

30
Ibid, p. 244

31
Persson & Savulescu, 2012, *op. cit*. note 2, p. 105.

32
Powell and Buchanan, op cit. note 6, p. 244.

33
Ibid, p. 245.

34
Ibid, p. 240.

35
Ibid, p. 256.

36
Ibid, p. 245.

37
Ibid, p. 245.

38
Ibid, p. 246.

39
S. Scheffler.1982. *The Rejection of Consequentialism*, Oxford, Oxford University Press.

40
Powell & Buchanan, *op. cit*. note 6, p. 253.

41
Ibid, p. 242.

42
Ibid, p. 247.

43
Ibid, p. 253.

44
Ibid, p. 253.

45
Ibid, p. 252.

46
Ibid, pp. 252–3.

47
Ibid, p. 242.

48
Persson & Savulescu, *op. cit*. 2012, note 2, p. 33–36.

49
Powell & Buchanan, *op. cit*. note 6, p. 242.

50
Ibid, p. 251.

51
Ibid, p. 250.

52
Persson & Savulescu, *op. cit*. 2012, note 2, p. 27

53
Powell & Buchanan, *op. cit*. note 6, p. 252.

54
Persson & Savulescu, *op. cit.* 2012, note 2, p. 30

55
Ibid: ch. 5.

56
Ibid: 26.

57
Ibid: 19ff.

58
Powell & Buchanan, *op. cit.* note 6, p. 250.

59
Ibid, p. 254.

60
Ibid, p. 255.

61
Ibid, p. 256.

62
Ibid, p. 249.

63
Ibid, p. 240.

64
Ibid, p. 251.

65
Persson & Savulescu, *op. cit*. 2012b, note 2, p. 105.

66
Powell & Buchanan, *op. cit*. note 6, p. 253.

67
Ibid, p. 258.

68
Persson & Savulescu 2014a, *op. cit* note 1; 2015b, *op. cit.* note 5.

69
Powell & Buchanan, *op. cit*. note 6; p. 256.

70
Ibid, p. 240: our emphasis.

